# Study of transpedicular screw fixation on spine development in a piglet model

**DOI:** 10.1186/s13018-015-0302-9

**Published:** 2016-01-13

**Authors:** Ya-min Shi, Fang-zheng Zhu, Xing Wei, Bing-yao Chen

**Affiliations:** Department of Orthopaedic Surgery, The First Affiliated Hospital of PLA General Hospital, NO. 51, Fu-chen road, Beijing, 100037 China; Department of Orthopaedic Surgery, The Second Artillery General Hospital of PLA, Beijing, 100048 China

**Keywords:** Transpedicular screw fixation, Spine development, A piglet model, Canalis vertebralis

## Abstract

**Background:**

Transpedicular screws may cause damage to the cartilage in the neural arch of the vertebra, and give continuous pressure to the skeleton besides the vertebral body. The aim of this study is to examine the morphological change of the vertebral body at fixation sites and development of the vertebral body after fixation.

**Methods:**

A piglet model was used to study the influence of transpedicular screw fixation on spine development. Transpedicular screw fixation was adjusted to meet specific requirements of surgery on piglet. The screws and plates were placed at L1–L3 vertebral plates via routine surgical approach. Scoliosis and kyphosis Cobb angles were measured.

**Results:**

Anatomical characteristics of 6-week-old piglets fit the transpedicular screw system, and can meet the requirements of related studies. Transpedicular screw fixation system has no significant influence on the development of canalis vertebralis. Fixation did not cause developmental stenosis of canalis vertebralis and damage to spinal cord or nerve root. However, transpedicular screw fixation significantly impacted the development of the spine: it shortened the spine by curtailing the length of the vertebral body and intervertebral space. Our results also suggested that slow growth of epiphyseal plate may contribute to the shortening of the vertebral body.

**Conclusion:**

Transpedicular screw fixation system is beneficial for fixation of the developing spine. It may not cause scoliosis but could lead to change of cervical curvature.

## Background

Transpedicular screw fixation was developed by Roy-Camille in the 1970s [1], and had been used to treat unstable thoracolumbar junction fractures. Since then, transpedicular screw fixation was widely used for spinal fracture, deformity, tumor, and degenerative diseases, boosting the development of spinal surgery [2–4].

Spinal deformity is a common disease with high frequency in adolescences [5]. As the understanding of spinal deformity increases, the importance of early treatment for spinal deformity had been noticed [6]. The processus spinosus and vertebral plate of children were frail, and the pedicle and vertebral body cannot form firm synostosis. Therefore, a planted instrument may not successfully fix and correct the deformity due to its weak stability. For children with spinal diseases such as spinal fraction, tumor, hemilaminectomy, or scoliosis correction, transpedicular screw fixation is the sole method to provide strong fixation [7]. This method had been widely used to treat spinal diseases of children under the age of 10 years.

Since spines of children are still in development, transpedicular screw fixation may slightly impact the development of spines. Jeszenszky et al. [8] demonstrated that after transpedicular screw fixation at L2, compared with L3, the diameter of the vertebral canal decreased by 5.4 %, and sagittal diameter decreased by 9.5 % in 6 months. Akin Cil et al. [9] also reported that after lateral transpedicular screw fixation, disorder of pedicle development and canales spinalis stenosis was found. However, these studies were limited to the analysis of a single screw on the neurocentral cartilage (NCC), and they did not include effects on spine development.

In this study, a piglet model was used to study the influence of transpedicular screw fixation on spine development. Transpedicular screw fixation was adjusted to meet specific requirements of operating on piglet. Animals were examined by CT and X-ray before and after surgery. The screws and plates were placed at L1–L3 vertebral plates through routine surgical approach. Data from this study suggested that transpedicular screw fixation system may be beneficial for fixation of developing spine, while not causing scoliosis.

## Materials and methods

### Animals

The use of piglets in this study is reviewed and approved by the ethical committee of General Hospital of People’s Liberty Army. Piglets (6-week-old, 9 males and 14 females, 8.5 ± 1.7 kg, 51 ± 4 cm in spine length) were used in this study. All animals passed the investigation by National Animal Quarantine Center, and the possibility of tumor and deformity of spine were ruled out by spiral computed tomography (CT) and X-ray.

### Group assignment

A cohort of 23 piglets were randomized into four groups: group A included three subjects as control, without any treatment; group B included three subjects as control, with vertebral lamina revelation (L1–L3); group C included five subjects as control, with six screws bilaterally placed at L1, L2, and L3 in each pig. All screws, which penetrated through cartilages in the neural arch of the vertebra, were left within pedicles; and group D included 12 subjects as treatment, with six screws bilaterally placed at L1, L2, and L3 in each pig. All screws were fixated with titanium plates.

All screws used in this study are made of titanium (Beijing Fule Technology, Inc. China). The dimensions of screws are 3.5 mm in diameter and 20 mm in length.

### Preparation before surgery

Preparation of pigs before surgery included the following: (1) spiral CT (GE LightSpeed Pro 32 Slice CT, General Electric, USA) and X-ray (RADREX general radiography system, Toshiba, Japan) investigation. (2) Preoperative fasting for 24 h. (3) Preoperative preserved skin: skins were shaved in corresponding areas (approximately 25 cm × 15 cm) then cleaned with soap water.

### Surgical procedure

Anesthesia: A mixture of 0.25 mL/kg Sumianxin and 18 mg/kg ketamine was used for anesthesia. The operation started as soon as corneal reflection disappeared. Depending on animal conditions, an additional anesthesia (half dose) might be used.

Positioning: Animals were at prone position with four limbs fixed.

Operation procedure: Skins at the incision site were sterilized with iodophor, and incised at the center of the back. The incision started from T14 to L4. Skins and subcutaneous tissues were cut layer by layer. Sub-periosteal dissection was conducted bilaterally, and paravertebral muscles were pulled aside. The sides of L1–L3 were revealed to the exterior side of zygopophysis, and the root of processus transversus were exposed.

Group B: After revelation, the incision sites were washed, sterilized with 70 % ethanol (volume/volume), and the wound was sutured. During the operation, approximately 15 mL of blood flowed out. The average time for surgery is about 1.5 h.

Group C: After revelation, a total of six screws were bilaterally placed into L1–L3. The average sagittal angle of L1–L3 pedicle and vertebral body is 42.5°. Thus, the screw sites were the central point on the root of processus transversus, with a separation angle of 40° between screws and vertical plane of spine (be perpendicular to vertebral lamina). After fixation, the incision sites were washed, placed with drainage strip, sterilized with ethanol, and sutured. During the operation, approximately 20 to 30 mL of blood flowed out. The average time for surgery was about 3 h.

Group D: After revelation, a total of six screws were bilaterally placed into L1–L3. The screw sites were the central point on the root of processus transversus, with a separation angle of 40° between screws and vertical plane of spine (be perpendicular to vertebral lamina). After the screws were placed, they were fixed with titanium plates. After fixation, the incision sites were washed, placed with drainage strip, sterilized with ethanol, and sutured. During the operation, approximately 20 to 30 mL of blood flowed out. The average time for surgery was about 3 h.

### Post-surgery procedure

Post-surgery procedures included the following: (1) sauteralgyl (50 mg) was administered through intramuscular injection. (2) Penicillin (800 K units) was administered through intramuscular injection, three times per day and for three consecutive days after surgery. (3) X-ray and spiral CT were used at different time points (1 day, 2 months, and 3 months after the operation). (4) Twelve days after the operation, stitches were removed. All animals were sacrificed 3 months after the operation.

### Post-surgery observation

**Fig. 1:**
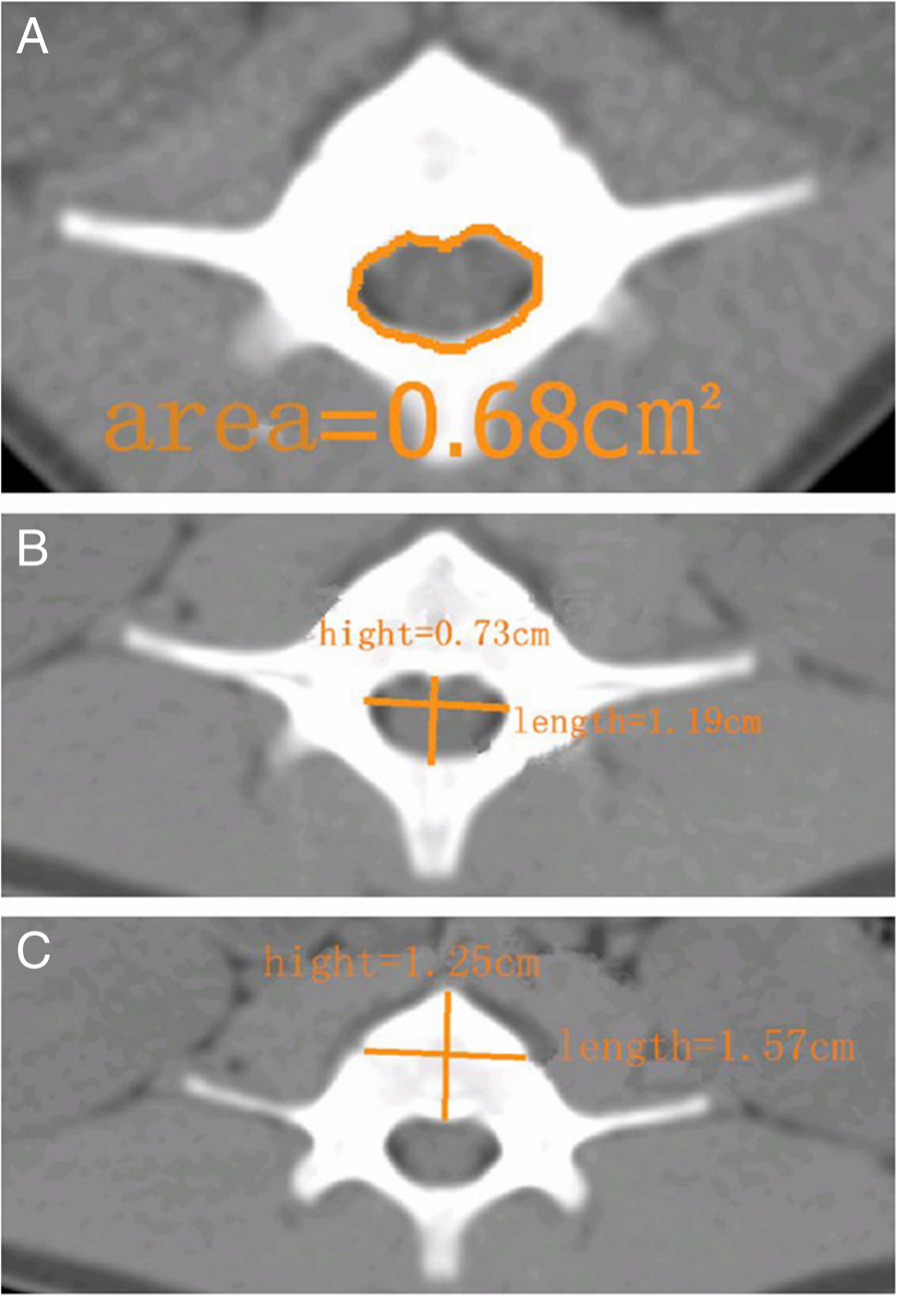
Images of CT are shown as examples of how the measurements of canal area (**a**), vertebral transverse/sagittal diameter (**b**), and canal transverse/sagittal diameter (**c**) were performed. Numbers in the images were typical readings for these measurements

**Fig. 2:**
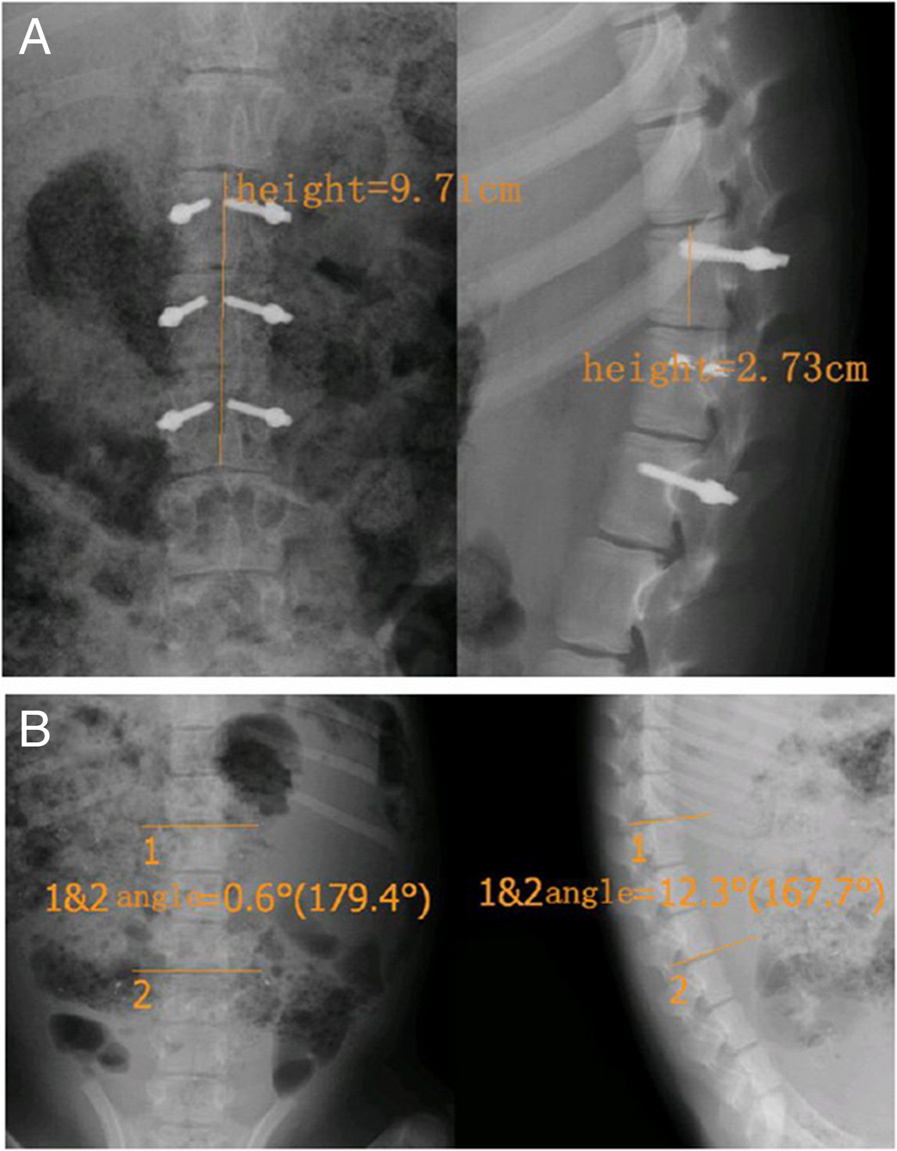
Images of X-ray are shown to illustrate how measurement of vertebral heights (**a**) and scoliosis/kyphosis Cobb angles (**b**) were conducted. Numbers in the images were typical readings for these measurements

**Fig. 3:**
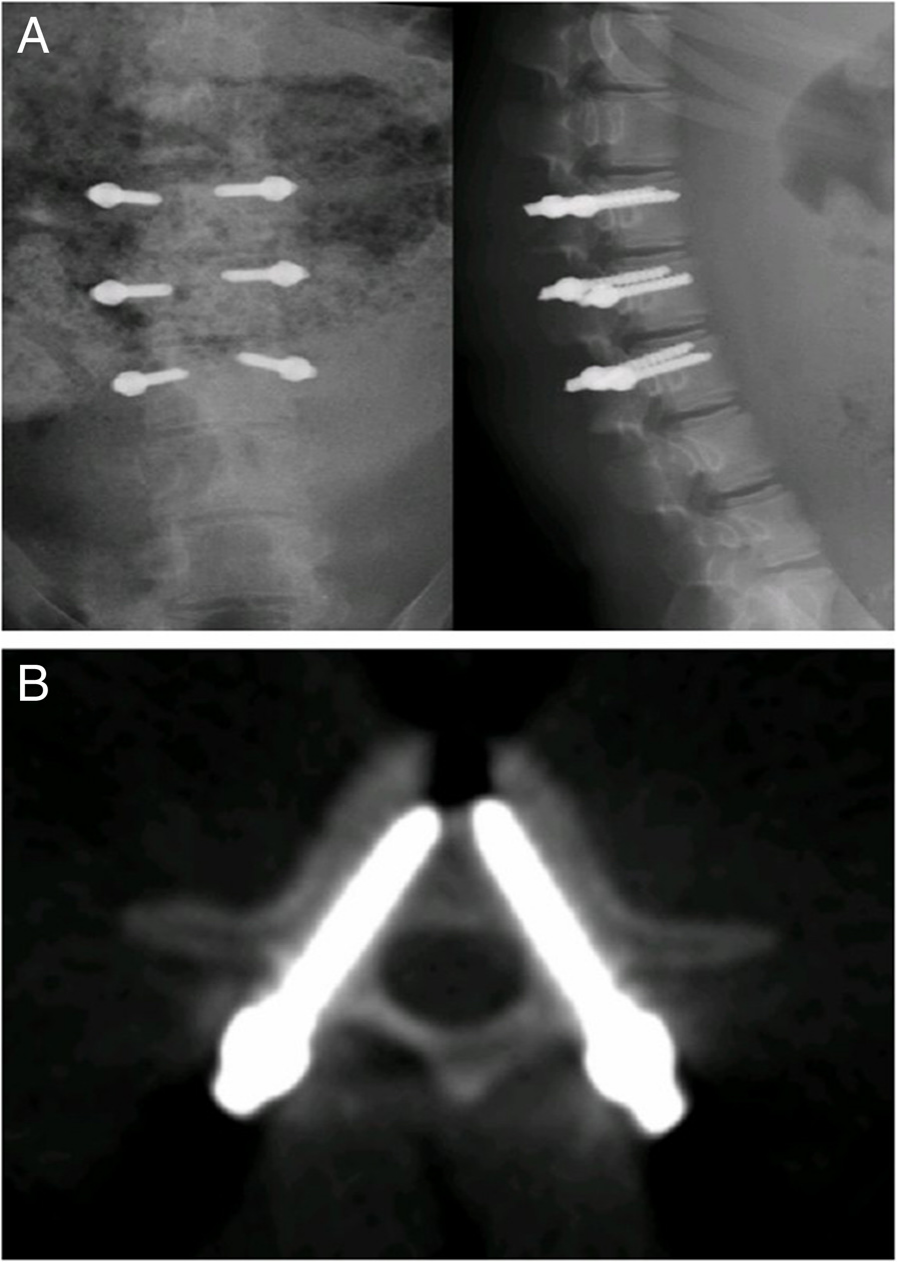
Lateral X-ray (**a**) and CT (**b**) examinations of pigs in group C were performed right after surgery. The *white shadow* indicated the implanted screws

X-ray and spiral CT were used for measurements (X-ray parameters 75 KV, 18 mAs, 90 ms; spiral CT parameters 120 KV, 100 mAs, 2.5 mm): (1) area, transverse diameter, and sagittal diameter of the vertebral canal (Fig. 1a); (2) transverse diameter, sagittal diameter, and height of the vertebral body (Fig. 1b); (3) spine lengths of L1–L3 (Fig. 1c).

The area of the vertebral canal was automatically measured by the software after drawing out its curve. The planes with the smallest area were chosen for further measurements of transverse diameter (the widest) and sagittal diameter of the vertebral canal, vertebral transverse diameter (the widest) and sagittal diameter, heights of the vertebral body, and spine lengths of L1–L3 (details shown in Fig. 2a).

Since no operations were conducted in group A, there were only one-time measurements by X-ray and spiral CT. Scoliosis and kyphosis of the spine were observed by the software affiliated with the imaging system (Fig. 2b). Stability of transpedicular screw fixation after implantation was also measured by using spiral CT and X-ray in a timely manner. Whether transpedicular screw fixation was mobilized or weakened was determined.

General observation: After sacrificing the piglets, the lumbar spine was taken out for further observation of scoliosis and kyphosis at transpedicular screw fixation sites.

### Histological analysis

Lumbar spine samples were decalcified with formic acid, imbedded with paraffin, sectioned, and stained with H&E. The morphology and arrangement of cartilage cells and change of intervertebral cartilages were observed under microscope. Spines of animals in group D were coronally and sagittally sectioned to observe the vertebral body change and intervertebral cartilages.

### Statistical analysis

All statistical analysis was performed with SPSS 12.0 software. Difference of vertebral heights and lengths of L1–L3 were analyzed by one-way ANOVA. *p* < 0.05 was considered as significant difference. If data was not suitable for one-way ANOVA, for example not normally distributed, then rank-sum test was used.

## Results

### General condition of animals

Due to overdosing in anesthesia (death from suffocation by glossocoma blocking the respiratory tract), one piglet in group D died before the operation and another died during the operation. In group C, infection occurred in two piglets after operation. The bacteria were identified as *Escherichia coli*. One animal was cured by disinfectant, the other died. The remaining 17 animals recovered from the operation without any infection or other morbidity (symptoms of spinal cord and nerve damage including limping, muscular dystrophy, neurogenic ulcer).Three months after the operation, the average weight was 40.4 ± 8.0 kg, while the average body length was 88.7 ± 7.3 cm.

### X-ray, spiral CT, and statistical analysis of spine

Area, transverse diameter, and sagittal diameter of the vertebral canal were measured. Also, transverse diameter, sagittal diameter, and height of the vertebral body were measured. In addition, spine lengths of L1–L3 were observed (Tables 1, 2, and 3).

**Table 1:** Summary of measurements on the vertebral body right after surgery (mm/mm^2^)

	Group A (*n* = 9)	Group B (*n* = 9)	Group C (*n* = 12)	Group D (*n* = 30)
Canal area	64.78 ± 8.07	63.89 ± 2.67	66.42 ± 14.79	64.03 ± 7.97
Canal transverse diameter	11.24 ± 0.78	11.31 ± 0.47	11.35 ± 1.46	11.72 ± 1.04
Canal sagittal diameter	7.16 ± 0.59	6.79 ± 0.35	7.13 ± 0.50	6.67 ± 0.61
Vertebral transverse diameter	17.02 ± 2.30	17.17 ± 0.46	17.18 ± 0.66	17.55 ± 1.81
Vertebral sagittal diameter	12.88 ± 1.54	12.50 ± 0.97	13.02 ± 1.14	12.06 ± 1.15
Vertebral height	15.67 ± 2.91	14.56 ± 1.03	16.03 ± 1.24	15.66 ± 2.08
L1–L3 length	45.93 ± 4.10	49.03 ± 3.01	56.48 ± 3.76	51.55 ± 6.27

**Table 2:** Summary of measurements on the vertebral body 3 months post-surgery (mm/mm^2^)

	Group A (*n* = 9)	Group B (*n* = 9)	Group C (*n* = 12)	Group D (*n* = 30)
Canal area	110.33 ± 14.67	112.89 ± 8.91	106.83 ± 14.10	107.63 ± 16.07
Canal transverse diameter	13.44 ± 1.80	13.52 ± 0.75	12.95 ± 1.14	13.53 ± 0.98
Canal sagittal diameter	9.59 ± 0.42	9.47 ± 0.78	8.88 ± 0.60	9.20 ± 0.86
Vertebral transverse diameter	22.76 ± 1.24	22.32 ± 1.24	22.86 ± 1.62	22.26 ± 2.20
Vertebral sagittal diameter	18.31 ± 1.25	18.47 ± 1.06	18.40 ± 1.31	17.18 ± 2.01
Vertebral height	28.39 ± 2.28	29.56 ± 0.92	29.17 ± 1.30	24.60 ± 3.45
L1–L3 length	93.57 ± 7.66	100.03 ± 3.12	94.55 ± 3.06	76.13 ± 4.39

**Table 3:** Vertebral heights of group D (mm)

	Right after surgery	3 months post-surgery
1	15.50 ± 2.19	25.85 ± 2.36
L2	15.48 ± 2.12	20.82 ± 1.73
L3	16.00 ± 2.11	27.12 ± 2.24

Right after the operation, there were significant differences in area, transverse diameter, and sagittal diameter of the vertebral canal; transverse diameter, sagittal diameter, and height of the vertebral body; and spine length of L1–L3 between groups (*p* > 0.05).

Three months after the operation, area, transverse diameter, and sagittal diameter of the vertebral canal were measured. Also, transverse diameter, sagittal diameter, and height of the vertebral body were measured. In addition, spine length of L1–L3 was observed.

There were no significant differences in area, transverse diameter, and sagittal diameter of the vertebral canal and transverse diameter, sagittal diameter, and height of the vertebral body between groups (*p* > 0.05). After implantation of transpedicular screw fixation system, area, transverse diameter, and sagittal diameter of the vertebral canal and transverse diameter, sagittal diameter, and height of the vertebral body did not decrease significantly.

There were significant differences in heights of the vertebral body and lengths of L1–L3 between groups A, B, C, and D (*p* < 0.05) while no significant difference within groups A, B, and C (*p* > 0.05). After implantation of transpedicular screws, heights of the vertebral body and spine lengths of L1–L3 did not change significantly. However, after fixation, heights of the vertebral body and spine lengths of L1–L3 significantly decreased.

There were no significant differences between heights of the vertebral body of L1 and L3 in group D (*p* > 0.05). However, the vertebral body of L2 is significantly shorter than that in L1 and L3 (*p* < 0.05).

### Scoliosis and kyphosis of the spine

**Fig. 4:**
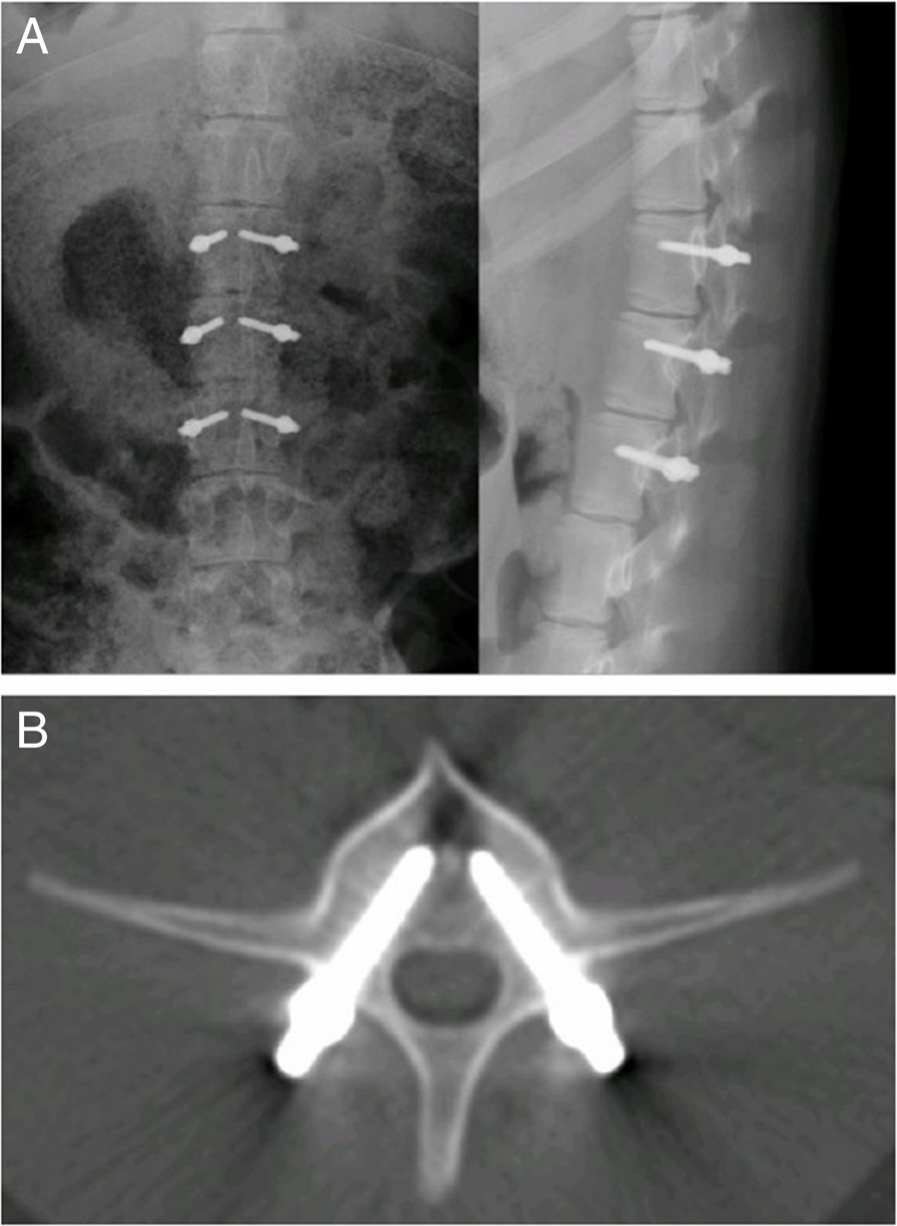
Lateral X-ray (**a**) and CT (**b**) examinations of pigs in group C were performed 3 months after surgery. The *white shadow* indicated the implanted screws

**Fig. 5:**
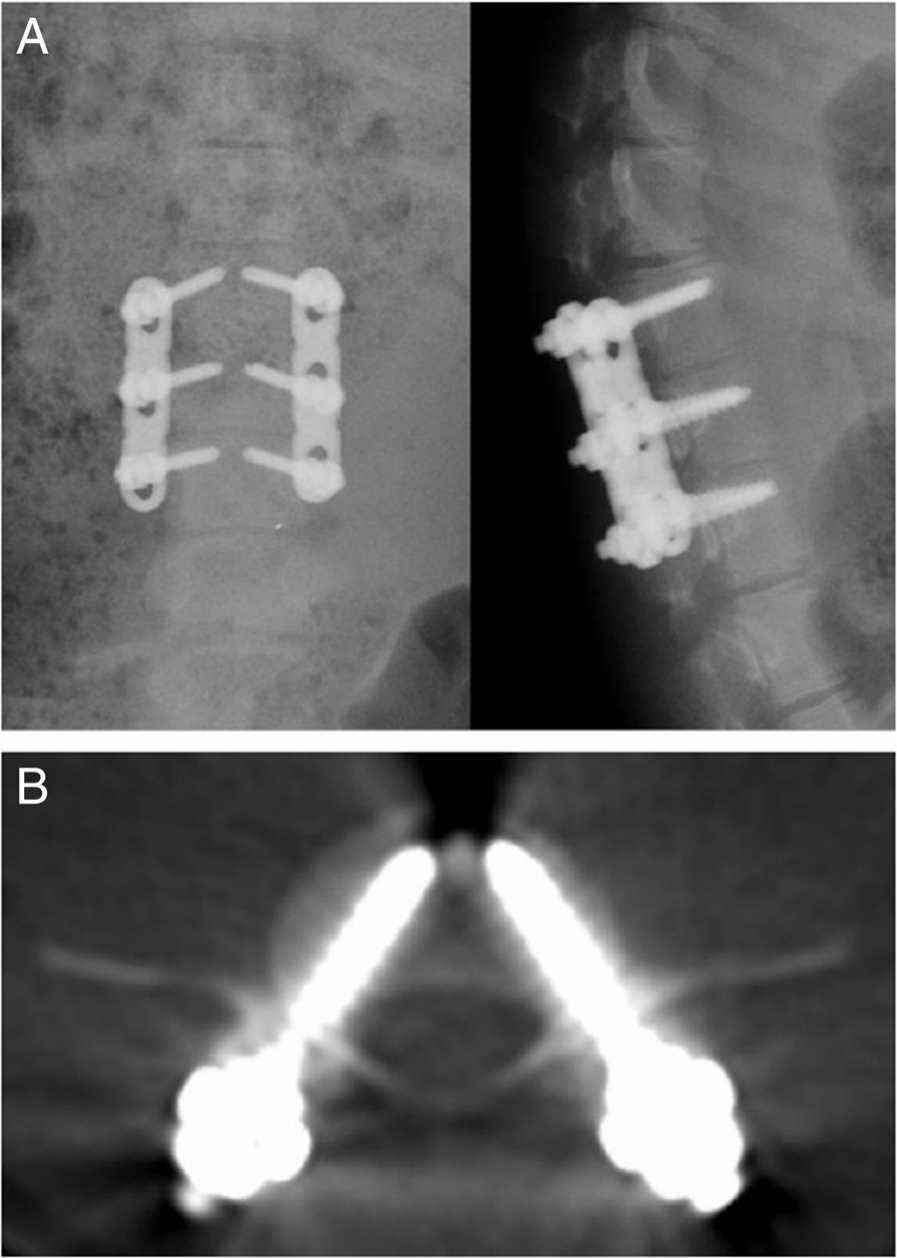
Lateral X-ray (**a**) and CT (**b**) examinations of pigs in group D were performed right after surgery. The *white shadow* indicated the implanted screws and plates

**Fig. 6:**
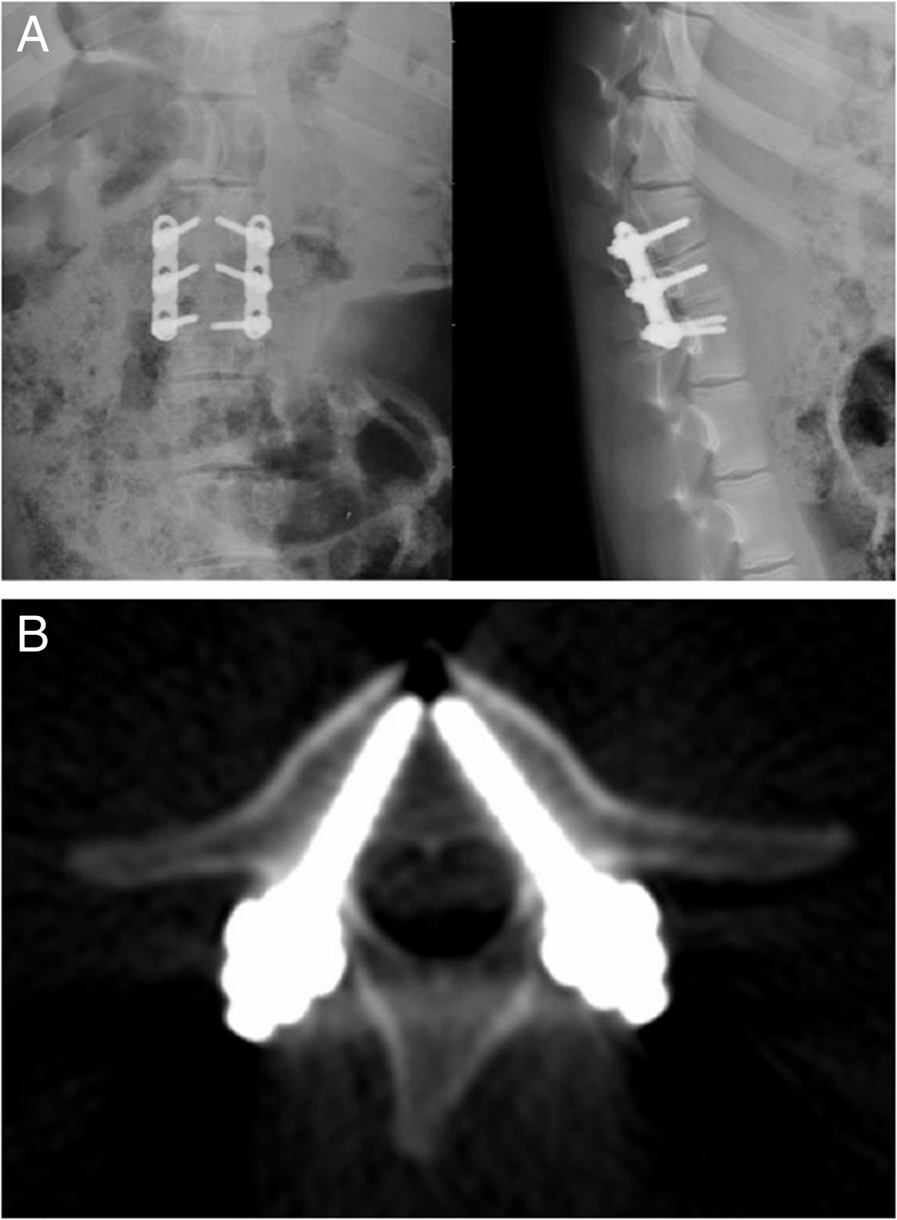
Lateral X-ray (**a**) and CT (**b**) examinations of pigs in group D were performed 3 months after surgery. The *white shadow* indicated the implanted screws and plates

Results of scoliosis of the spine are listed in Table 4 and shown in Figs. 3a, 4a, 5a, and 6a. There was no obvious scoliosis, and the Cobb angles were relatively small in all animals. Cobb angle is defined as the angle formed between a line drawn parallel to the superior endplate of one vertebra above the fracture and a line drawn parallel to the inferior endplate of the vertebra one level below the fracture. These results indicated that transpedicular screw fixation had no significant impacts on cervical curvature of coronal plane of the lumbar spine.

**Table 4:** Scoliosis Cobb angle of piglets before and after surgery (°)

	Before surgery	Right after surgery	3 months after surgery
A group			
A_1_	0.6	0.6	1.4
A_2_	4.6	4.6	2.1
A_3_	1.3	1.3	2.4
B group			
B_1_	0.5	0.4	1.3
B_2_	0.7	0.8	1.0
B_3_	1.7	2.1	0.2
C group			
C_1_	0.8	2.0	3.5
C_2_	1.5	0.8	4.2
C_3_	0.3	2.8	1.2
C_4_	2.9	0.2	1.1
D group			
D_1_	0.6	0.7	0.8
D_2_	1.7	4.0	1.9
D_3_	1.0	1.4	1.3
D_4_	1.8	2.0	0.3
D_5_	2.1	1.0	3.2
D_6_	1.8	1.0	1.1
D_7_	0.9	1.0	2.5
D_8_	0.6	0.5	7.9
D_9_	0.6	4.6	1.8
D_10_	1.8	0.0	0.6

The results of kyphosis of the spine are listed in Table 5 and shown in Figs. 3a, 4a, 5a, and 6a. Transpedicular screw fixation had no apparent impacts on cervical curvature of sagittal plane of the lumbar spine since the spines of animals in groups A, B, and C kept a kyphotic curvature before and after operations. Before the operation, spines of animals from group D kept a kyphotic curvature which remarkably decreased after the operation. In three piglets, it even turned into slight lordosis. Three months after the operation, lordosis of spine in seven piglets became more obvious, while the remaining three piglets stayed kyphotic in curvature. In two animals, the kyphotic curvature decreased significantly while it slightly increased in one pig after the operation. These results indicated that transpedicular screw fixation had significant impacts on cervical curvature of the lumbar spine.

**Table 5:** Kyphosis Cobb angle of piglets before and after surgery (°)

	Before surgery	Right after surgery	3 months after surgery
A group			
A_1_	3.4	3.4	10.9
A_2_	13.8	13.8	8.5
A_3_	6.9	6.9	8.3
B group			
B_1_	9.4	9.7	6.9
B_2_	11.3	10.5	3.3
B_3_	9.7	9.8	1.2
C group			
C_1_	2.4	11.9	16.7
C_2_	18.1	14.8	9.9
C_3_	18.0	21.9	23.0
C_4_	17.0	18.9	5.0
D group			
D_1_	12.6	0.5	−3.7
D_2_	18.6	3.4	−4.2
D_3_	15.7	2.7	0.6
D_4_	10.5	3.9	−4.3
D_5_	17.5	−2.9	−0.7
D_6_	9.2	−0.7	−3.8
D_7_	9.1	−2.5	6.3
D_8_	11.3	10.3	−6.9
D_9_	11.3	7.0	−2.1
D_10_	18.1	3.7	0.8

All positive values correspond to kyphosis angle. All negative value correspond to lordosis angle

### Observation of transpedicular screw fixation system in implanted animals

A total of 24 screws were implanted into four piglets in group C. Three months after the operations, X-ray and spiral CT were used to investigate the animals on a monthly basis. There were no mobilization, pullout, and fracture of screws (Figs. 3a, b and 4a, b).

There were 60 screws implanted into ten piglets from group D. Three months after the operations, X-ray and spiral CT were used to investigate the animals on a monthly basis. There were no pullout and fracture of screws. However, the mobilization of screws was apparent, especially for L2, in which screws moved from the front to the center (Figs. 5a, b and 6a, b).

### Observation of lumbar vertebrate after sacrifice

**Fig. 7:**
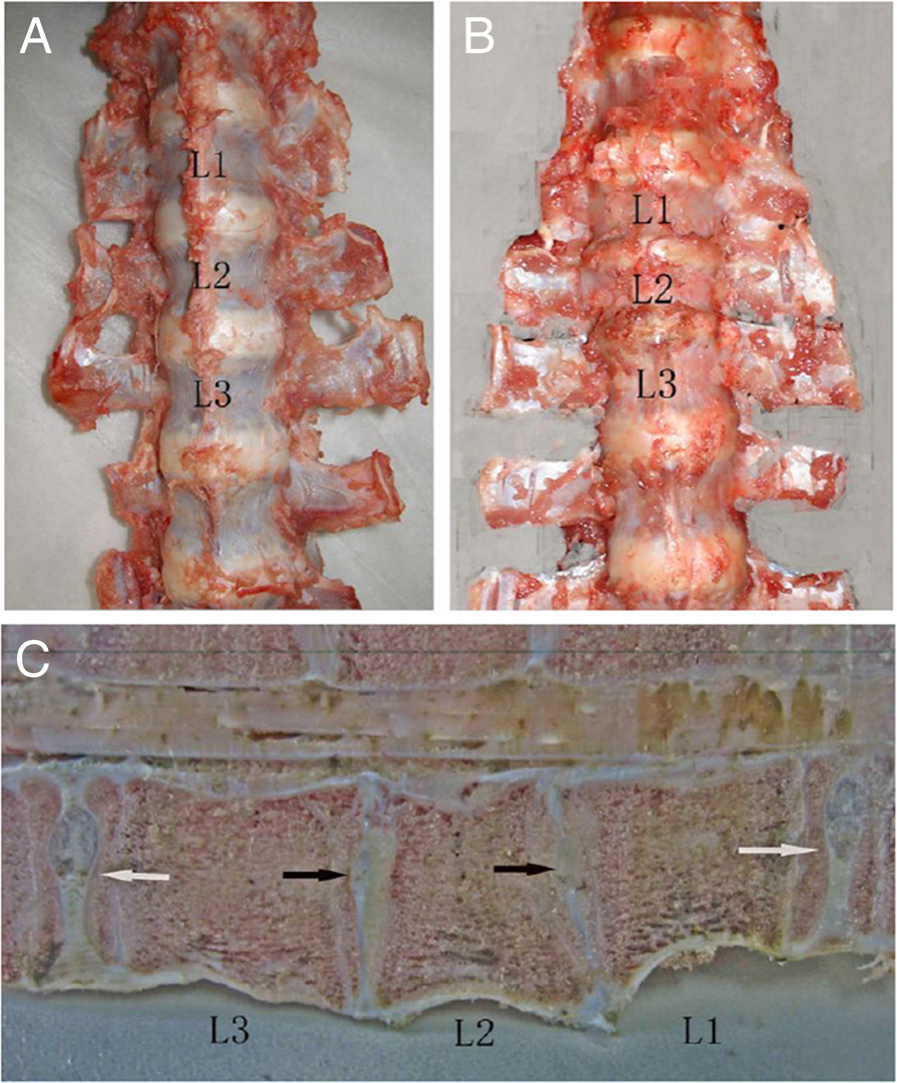
Gross-looking pig spine was examined 3 months post-surgery. **a** Overview of group A. **b** Overview of group D. **c** Sagittal profile image of group D. *White arrows* indicate fixed segment intervertebral. *Black arrows* indicate adjacent non-fixed segment intervertebral

No abnormality was observed in groups A and B. There was no spontaneous fusion of vertebral plates behind L1–L3 (Fig. 7a).

Group C: There was no mobilization or pullout of screws, which were covered by bone tissues and conjunctive tissues. Also, there was no spontaneous fusion of vertebral plates behind L1–L3. Additionally, no abnormality in vertebral plates and intervertebral cartilages was observed.

Group D: There was no mobilization or pullout of screws, which were covered by bone tissues and conjunctive tissues. Also, there was no spontaneous fusion of vertebral plates behind L1–L3. At fixation sites, intervertebral space was extremely narrow and intervertebral cartilages were thin. There was almost no nucleus pulposus, while the vertebral body of L2 had a slightly morphological change. However, there was no fusion in intervertebral space, confirmed by the X-ray results (Fig. 7b, c).

### Histological analysis

#### The morphological change in annular epiphysis of the vertebral body

**Fig. 8:**
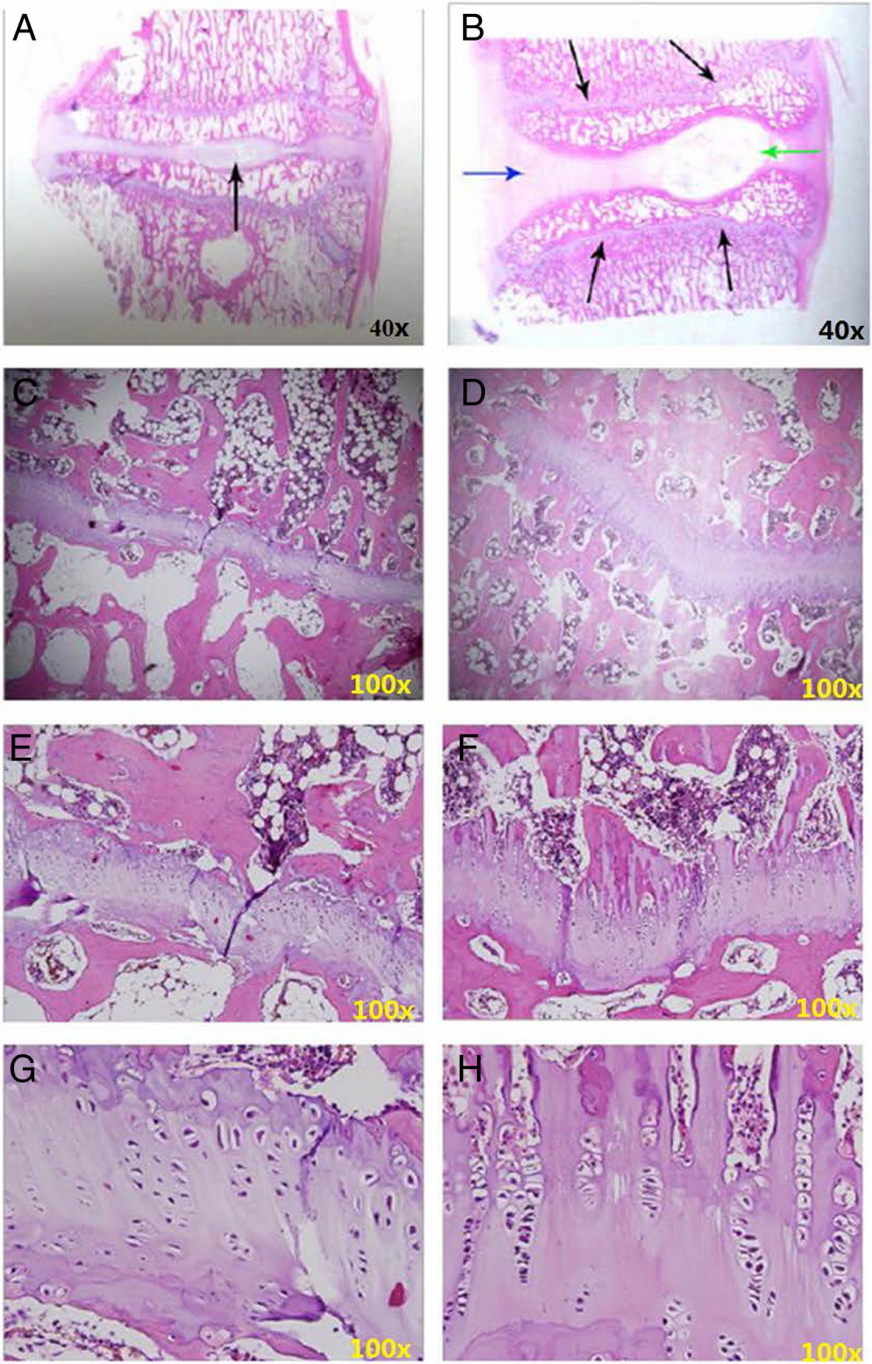
H&E staining showed annular epiphysis in group A and group D 3 months post-surgery. **a** A representative image of epiphysis from group D is shown. A *black arrow* indicates the nucleus. **b** A representative image of epiphysis from group A is shown. *Black arrows* indicate the epiphyseal plate. A *blue arrow* indicates the disc. A *green arrow* indicates the nucleus. **c**, **e**, **g** Enlarged images of epiphysis from group D. **d**, **f**, **h** Enlarged images of epiphysis from group A. *Numbers* in the left-lower corner indicate the image’s magnification

Judging from the sagittal plane of the lumbar spine, epiphyseal plates of group D were shorter than their counterparts in control groups. Figure 8a shows that annular epiphysis was stained positive for hematoxylin which is an indication of glycosaminoglycan accumulation. At fixation sites, annular epiphysis chondrocytes were morphologically abnormal, since they were smaller and contained more cytoplasm than normal cells. Also, alignment of chondrocytes at fixation site was abnormal comparing to normal tissue. Trabecular besides epiphyseal plates were thin and small (Fig. 8a–h).

#### The change of intervertebral cartilages

**Fig. 9:**
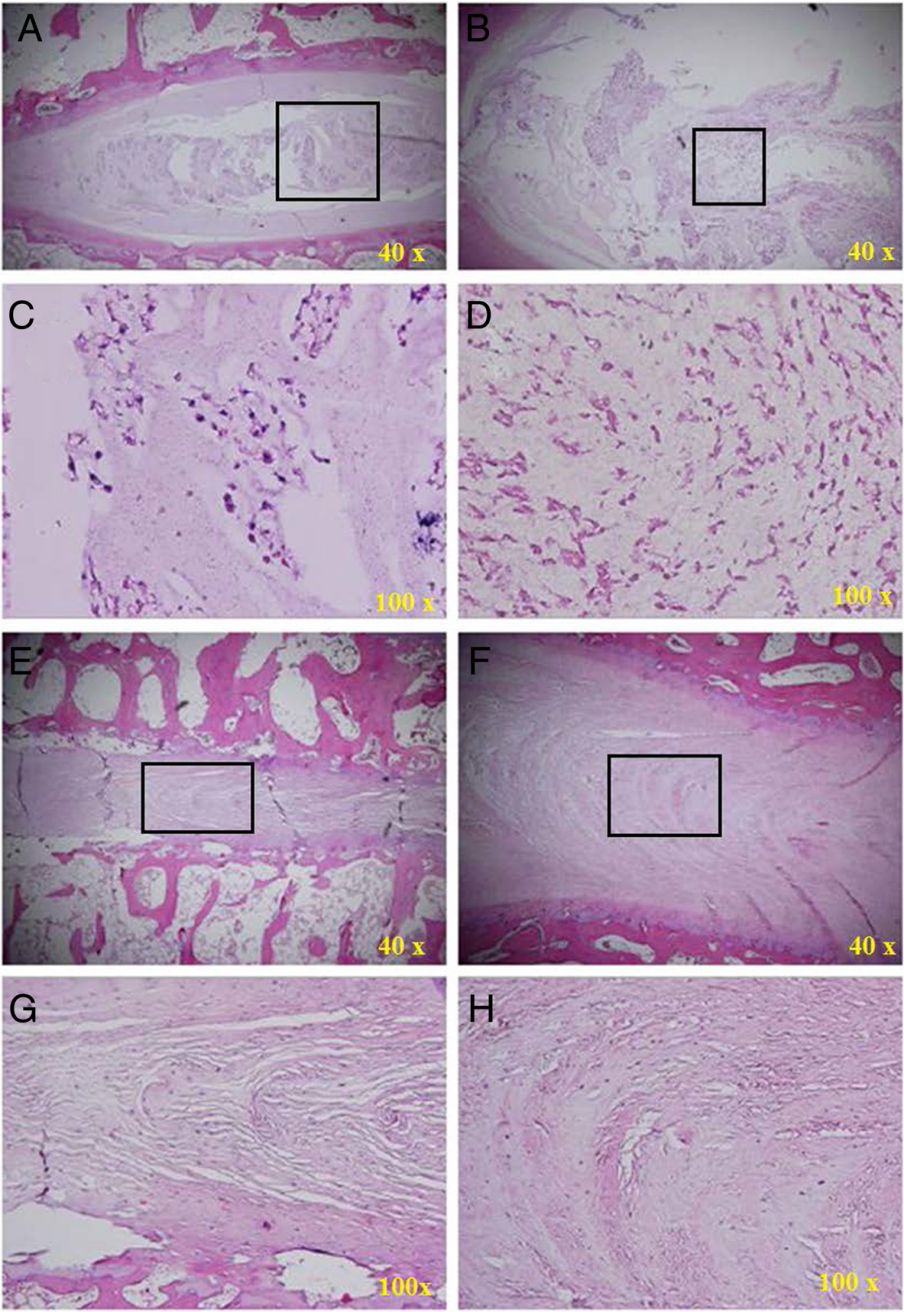
H&E staining shows nucleus and annulus in group A and group D 3 months post-surgery. **a** A representative image of nucleus from group D. **b** A representative image of nucleus from group A. **c** Enlarged image of *boxed area* in upper panel. **d** Enlarged image of *boxed area* in upper panel. **e** A representative image of annulus from group D. **f** A representative image of annulus from group A. **g** Enlarged image of *boxed area* in upper panel. **h** Enlarged image of *boxed area* in upper panel. *Numbers* in the left-lower corner indicate the image’s magnification

Judging from the sagittal plane of the lumbar spine, the intervertebral cartilage of group D was significantly shorter than their counterparts in control groups (Fig. 8a); nucleus pulposus was significantly smaller and contained less chondrocytes and hyaline tissue than in controls (Fig. 9a–d); annulus fibrosus became thinner with increasing number of chondrocytes (Fig. 9e–h).

#### The interphase between bone and screws

**Fig. 10:**
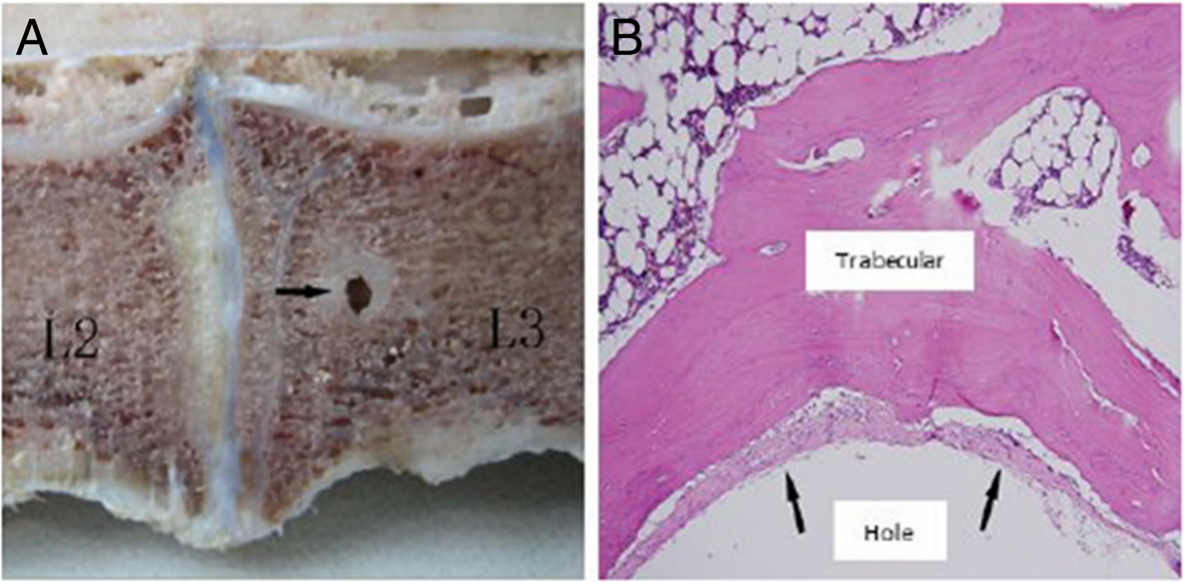
Screw hole in group D. **a** Sagittal profile image of group D. A *black arrow* indicates the screw hole. **b** H&E staining shows tissue surrounding screw hole. ×100 magnification

We observed that there were white non-bone tissues surrounding the screws (Fig. 10a). Through H&E staining, it was shown that they were fibrotic tissues, surrounded by trabecular bone (Fig. 10b). These results indicated that screws and bone tissues were weakly connected with thick parenchyma.

## Discussions

This study was performed to investigate the influence of transpedicular screw fixation on piglet lumbar spine through measurements of piglets’ spine. The screws and plates were placed at sides of L1–L3 vertebral plates through routine surgical approach from the central back.

### The impacts of transpedicular screw fixation on the development of the vertebral canal

It has been reported that the fixation system could not lead to developmental stenosis of the vertebral canal because its morphology had been fully developed after 1 year old [10]. In addition, Zindrick et al. [11] showed that the vertebral canal could not further grow after 3 years old. Ruf and Harms [12] reported that with over 6-year follow-up studies of 16 children (ages 1 to 2) with transpedicular screw fixation, there was no stenosis of the vertebral canal, concurrent neurological diseases, or disorder of spinal cord development. Another clinical study from them [13] further confirmed these results. Our results also confirmed that no stenosis of the vertebral canal or neurological damage occurred due to morphological change of the spine at fixation sites.

This study showed that transpedicular screw fixation had no impacts on vertebral canal development. This could be explained by the following reasons: (1) the vertebral canal of piglets (6 weeks old) was mostly developed in terms of morphology. (2) Three months after the operation, transverse diameter and sagittal diameter of the vertebral canal in groups A, B, and C increased only 2.0 and 2.3 mm on average, respectively. The external diameter of screws was only 3.5 mm, approximately half of the diameter of pedicles. Also, the internal diameter of screws was only 2.5 mm, posing little damage to NCC. (3) As the spine of piglets develops, the diameter and length of screw decreased relatively, providing less holding strength. Thus, the impacts of screws further decreased. (4) During the development of the vertebral canal, the relative position of screws and spine changed. The distance between screws and pallium in the front area of the vertebral body 3 months after the operation and right after the operation was compared using the X-ray results. The fixation effects further decreased while the spine developed continuously.

Based on these four reasons, transpedicular screw fixation had limited impacts on sagittal diameter of the vertebral canal. In this study, no transversal junctions were used for spine fixation. Therefore, the transversal development of the vertebral canal was not hindered.

### The impacts of transpedicular screw fixation on development of the vertebral body and fibrocartilagines intervertebrales

To animals with developing spines, transpedicular screw fixation can affect the development of the vertebral body and intervertebral cartilages, when providing firm holding strength. Transpedicular screw fixation can limit the development of the vertebral body and also leads to dysplasia of intervertebral cartilages. Since the limitation of fixation system, the vertical growth of the vertebral bodies was hindered within the limited heights of intervertebral cartilages. Therefore, intervertebral space at fixation sites showed stenosis to different extents. Moreover, from the X-ray image of the lumbar spine, it was almost impossible to observe the intervertebral space 3 months after the operation. Based on these analyses, transpedicular screw fixation controlled the spine development through limiting the vertical growth of the vertebral body. If the direction of gravity was taken into account, the limitation of transpedicular screw on vertical growth of the vertebral body could be more obvious, since the direction of gravity is different in humans comparing to pigs. On the other hand, the transpedicular screw combined with plate may give enough support to counteract gravity; thus, the limitation of transpedicular screw on growth of the vertebral body may not be strengthened by gravity. It is hard to predict the influence of gravity on growth of the vertebral body with the data we currently have. To answer this question, further studies must be conducted.

However, our data also showed that the growth of the vertebral body at fixation sites, especially L2, did not completely cease. This phenomenon might be explained by the following reasons: (1) the relative length of screws decreased as the spine developed, resulting in less holding strength to the spine; (2) due to the vertical growth of spines, screws showed cutting function within sclerotin; (3) due to the continuous stress from transpedicular screw fixation, the connections between screws and titanium plates deformed. Therefore, based on the aforementioned reasons, transpedicular screw fixation could not fully limit the development of spines.

### Histological analysis of impacts of transpedicular screw fixation on spine development

Through sagittal sections of lumbar spine samples, the changes in intervertebral cartilages were observed in this study. It was demonstrated that chondrocytes in epiphysis were sensitive to mechanical pressure [14]. Due to mechanical pressure, the number, arrangement, and morphology of chondrocytes could change significantly, which disturbed the function of chondrocytes and affected the height and morphology of vertebral bodies. Our observation from H&E staining on annular epiphysis was in line with these previously reported results.

This study investigated the effects of transpedicular screw fixation on spine development in 20 piglets, and laid a solid foundation for its clinical application in treating children’s spinal diseases. Admittedly, this work is still a pilot study due to a small number of subjects.

## Conclusions

Transpedicular screw fixation system had no significant influence on canalis vertebralis development, and neither did it cause developmental stenosis of canalis vertebralis nor damage of spinal cord and nerve root. Transpedicular screw fixation system could significantly shorten the spine by shortening the length of the vertebral body and intervertebral space. It was shown that slow growth of epiphyseal plate could contribute to the shorter length of the vertebral body. Transpedicular screw fixation system is beneficial for the development of the spine. It will not cause scoliosis but can lead to change of cervical curvature.

## References

[1] Roy-Camille R (1989). Current trends in surgery of the spine. Int Orthop.

[2] Zindrick MR (1991). The role of transpedicular fixation systems for stabilization of the lumbar spine. Orthop Clin North Am.

[3] Van Brussel K, Vander Sloten J, Van Audekercke R, Fabry G (1996). Internal fixation of the spine in traumatic and scoliotic cases. The potential of pedicle screws. Technol Health Care.

[4] Spivak JM, Balderston RA (1994). Spinal instrumentation. Curr Opin Rheumatol.

[5] Lonstein JE, Winter RB, Moe JH, Bradford DS, Chou SN, Pinto WC (1980). Neurologic deficits secondary to spinal deformity. A review of the literature and report of 43 cases. Spine (Phila Pa 1976).

[6] McMaster MJ, Ohtsuka K (1982). The natural history of congenital scoliosis. A study of two hundred and fifty-one patients. J Bone Joint Surg Am.

[7] Modi HN, Suh SW, Fernandez H, Yang JH, Song HR (2008). Accuracy and safety of pedicle screw placement in neuromuscular scoliosis with free-hand technique. Eur Spine J.

[8] Jeszenszky D. Morphological changes of spinal canal after placement of pedicle screws in new pigs. *Scoliosis Research Society Annul Meeting*. 2000. Cairns: Australia.

[9] Cil A, Yazici M, Daglioglu K, Aydingoz U, Alanay A, Acaroglu RE (2005). The effect of pedicle screw placement with or without application of compression across the neurocentral cartilage on the morphology of the spinal canal and pedicle in immature pigs. Spine (Phila Pa 1976).

[10] Wild A, Jager M, Koch H, Webb JK (2002). Treatment of congenital scoliosis in an 8-month-old child. Arch Orthop Trauma Surg.

[11] Zindrick MR, Knight GW, Sartori MJ, Carnevale TJ, Patwardhan AG, Lorenz MA (2000). Pedicle morphology of the immature thoracolumbar spine. Spine (Phila Pa 1976).

[12] Ruf M, Harms J (2002). Pedicle screws in 1- and 2-year-old children: technique, complications, and effect on further growth. Spine (Phila Pa 1976).

[13] Ruf M, Harms J (2003). Posterior hemivertebra resection with transpedicular instrumentation: early correction in children aged 1 to 6 years. Spine (Phila Pa 1976).

[14] Buckwalter JA, Mower D, Ungar R, Schaeffer J, Ginsberg B (1986). Morphometric analysis of chondrocyte hypertrophy. J Bone Joint Surg Am.

